# Evaluation and treatment of failed shoulder instability procedures

**DOI:** 10.1007/s10195-016-0409-8

**Published:** 2016-06-15

**Authors:** Anthony G. Ho, Ashok L. Gowda, J. Michael Wiater

**Affiliations:** Department of Orthopaedic Surgery, Beaumont Health, 3535 W. Thirteen Mile Rd, Suite 744, Royal Oak, MI 48073 USA

**Keywords:** Failed, Instability, Shoulder, Evaluation, Treatment

## Abstract

Management of the unstable shoulder after a failed stabilization procedure can be difficult and challenging. Detailed understanding of the native shoulder anatomy, including its static and dynamic restraints, is necessary for determining the patient’s primary pathology. In addition, evaluation of the patient’s history, physical exam, and imaging is important for identifying the cause for failure after the initial procedure. Common mistakes include under-appreciation of bony defects, failure to recognize capsular laxity, technical errors, and missed associated pathology. Many potential treatment options exist for revision surgery, including open or arthroscopic Bankart repair, bony augmentation procedures, and management of Hill Sachs defects. The aim of this narrative review is to discuss in-depth the common risk factors for post-surgical failure, components for appropriate evaluation, and the different surgical options available for revision stabilization.

*Level of evidence* Level V.

## Introduction

The shoulder is the least constrained joint in the body, and is therefore susceptible to high rates of instability. In the United States, the incidence of shoulder dislocations is 23.0 per 100,000 person-years, with the highest rates in adults in their 20s [1].

Because the glenohumeral joint is vulnerable to symptomatic recurrence after a traumatic dislocation, surgical repair is often advocated. Traditionally, open repair has been the gold standard for stabilization; however, with newer methods and implants, arthroscopic repair is now preferred. Numerous studies over the past decade have shown equivalent outcomes between these two modalities [2].

Despite improvement in outcomes following primary stabilization surgery, a 3–25 % instability recurrence rate presents the most challenging post-surgical complication [3–5]. This review serves to analyze the causes for failure, appropriate evaluation, and treatment options when considering revision surgery for failed surgical stabilization.

## Anatomy

### Soft tissue

Glenohumeral joint stability is achieved through a combination of static and dynamic components. The rotator cuff serves as the main dynamic stabilizer, providing compression of the humeral head against the glenoid concavity, centering it during range of motion. Rotator cuff tears can result in uncoupling of these balanced forces across the joint, resulting in instability.

Static stability is maintained by the labral complex and the capsuloligamentous structures. The labrum consists of fibrocartilagenous tissue that lines the rim of the glenoid, and serves several functions. First, it increases the surface area of the glenoid and deepens the socket by 50 %, thereby providing a “bumper” effect along the bony periphery [6]. Second, and more significantly, it provides a strong anchor point for the capsular ligaments, particularly the anterior band of the inferior glenohumeral ligament. The classic Bankart lesion involves avulsion of the anteroinferior labrum off of the glenoid during anterior shoulder dislocations, resulting in destabilization of these protective mechanisms. Uhorchak et al. [3] reported that 68 % of patients with recurrent dislocations had a standard Bankart lesion, while 18 % had other labral abnormalities, including tearing, degeneration, or fraying. Sisto et al. [7] showed even higher rates of Bankart lesions associated with instability.

### Bony stability

**Fig. 1:**
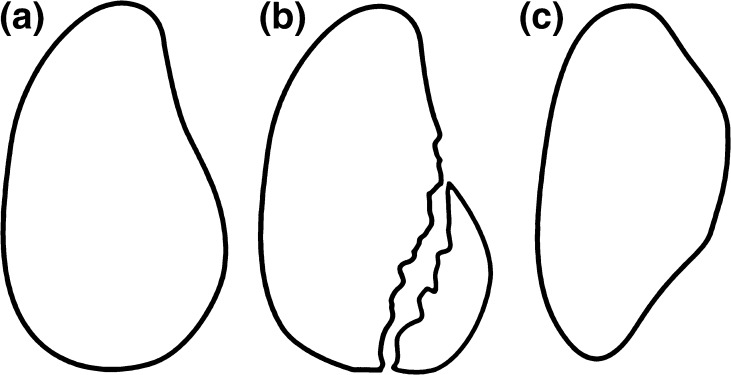
The normal glenoid morphology is pear shaped (**a**). With loss of the anterior glenoid rim (**b**), the glenoid takes on an inverted pear shaped morphology (**c**) [9]

Soft tissue stability is complemented by the bony structure of the glenoid and humeral head. When viewed frontally, the glenoid has a pear shape, with the inferior half wider than the superior half [8]. With recurrent anterior dislocations, two types of osseous defects can result. In the first, attritional loss of the anterior-inferior aspect of the glenoid results from repetitive wear and erosion. Burkhart and DeBeer [9] described this as the “inverted pear” appearance (Fig. 1). Alternatively, a bony Bankart lesion may result, in which a separate osseous fragment fractures from the glenoid.

In a cadaveric study of eight shoulders with step-wise osseous defects, Itoi et al. [10] identified 21 % anterior-inferior bone loss as the threshold for increasing anterior instability. These results have been corroborated in clinical studies, as high rates of failure have resulted when osseous deficiency was not appreciated or under-corrected [9].

Shoulder stability can also be compromised by failure to recognize humeral head defects. Hill Sachs lesions occur when the soft, posterolateral aspect of the humeral head impacts on the cortical rim of the anterior glenoid during dislocation. Incidence ranges from 70 % in first time dislocators to 100 % in recurrent dislocators [9, 11]. A study by Kaar et al. [12] determined that defects greater than 5/8 radius of the humeral head resulted in increased instability, whereas those smaller did not. Burkhart and De Beer [9] suggested defect orientation was more important, differentiating “engaging” from “non-engaging” Hill Sachs lesions.

Arciero et al. [13], in a 3-D modeling study, explored the cumulative effect on instability when both glenoid and humeral head defects were present. They found that simultaneous lesions had an additive effect on instability, and cautioned that isolated Bankart repairs may be insufficient in these situations.

## Causes for failure

When managing recurrent instability after a failed procedure, identification of the specific cause of failure is imperative before planning treatment course (Table 1). Investigation is necessary to avoid repeating potential errors and to appropriately educate the patient on risk of future recurrences.

**Table 1:** Risk factors for recurrence after Bankart repair

Recurrent trauma	
Patient factors	Younger age
	Male sex
	Increased number of dislocations
	Prior procedures
Missed diagnoses	Anterior glenoid defect
	Hill Sachs defect
	HAGL lesion
	Capsular laxity
Technical errors	Medial placement of glenoid anchors
	“High” placement of inferior glenoid anchors
	Insufficient number of anchors
	Improper suture configuration

### Recurrent trauma

Traumatic injuries to the surgically repaired shoulder are one of the biggest contributors to recurrence. As the majority of those affected are young with initial injuries often due to athletic activities, return to sport predisposes this population to re-injury. Tauber et al. [14] reviewed 41 patients and found that 85 % of initial shoulder dislocations and 59 % of re-dislocations after surgical stabilization were due to trauma.

### Patient factors

Age and sex have been strongly correlated with instability recurrence after primary stabilization. In a study of over 5900 patients, those younger than 20 years of age had a 12.6 % risk of postoperative dislocation and a 7.7 % revision rate after primary stabilization, compared to rates of 5.5 % and 2.8 %, respectively, in patients older than 29 years of age [15]. When compared to adults, younger patients are predisposed due to their higher activity level, more compliant tissue, and decreased muscle bulk [16, 17]. In addition, male patients are also at higher risk when compared to females. In one study, 90 % of patients with recurrent dislocations after arthroscopic repair were male [18].

The number of prior dislocations, in addition to the number of previous surgeries, negatively correlates with post-surgical success [16, 19]. In a study by Wasserstein et al. [15], patients with three or more dislocations had double the risk for revision surgery and ten times the risk of re-dislocating. In a separate analysis, patients with more than one stabilization procedure trended toward lower functional outcomes and patient satisfaction [20]. These results likely stem from progressive damage to the tissue, with diminished bone and soft tissue quality.

### Unaddressed glenoid defect

The relatively high incidence of anterior glenoid defects has been documented in the literature. In a study of 41 patients undergoing revision surgery, 51 % had a bony Bankart lesion greater than 2 mm, and an additional 5 % had an “eroded” anterior rim [14]. Initial outcomes with attempted soft tissue repair in patients with glenoid deficiency have not been promising. Burkhart and De Beer [9] described their suboptimal results after arthroscopic Bankart repair for “inverted-pear” bony glenoid defects. Patients without significant bony deficiency had only a 4 % recurrence rate, whereas those with bony loss had a 67 % recurrence. The authors determined that such patients were poor surgical candidates for soft tissue repair alone.

On the other hand, Mologne et al. [21] analyzed patients with anteroinferior glenoid bone deficiency ranging from 20 % to 30 % that were stabilized by arthroscopic Bankart repair. They suggested that arthroscopic repair was a good option when a bony glenoid piece could be incorporated, but that repairs with attritional loss were still less predictable. This was supported by a systematic review, which showed no significant recurrence increase when a bony fragment was identified, but inferior results when defects were due to erosion [16].

More recently, Yamamoto et al. [22] proposed the popular concept of the glenoid track. This model evaluated the relationship of the anterior glenoid rim to the medial margin of the Hill Sachs lesion in various positions and may better account for both humeral and glenoid defects when attempting to predict instability. A study by Giacomo et al. [23] proposed a treatment algorithm based on the degree of this bipolar bone loss.

### Humeral head defect

**Fig. 2:**
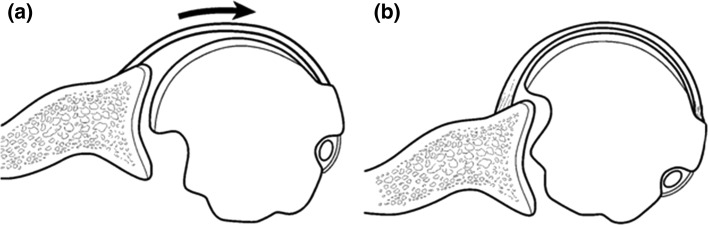
As the arm externally rotates in abduction, large Hill Sachs lesions in the posterior-superior humeral head (**a**) can engage the anterior glenoid rim, resulting in symptoms of instability even in the absence of a Bankart lesion (**b**) [9]

Hill Sachs lesions contribute to the risk of glenohumeral instability by shortening the rotational arc length of the humeral head on the glenoid. As the arm progressively abducts and externally rotates, large defects can engage and pivot the head on the anterior glenoid rim, causing a subluxation or dislocation event. Burkhart and DeBeer [9] were the first to describe the concept of Hill Sachs “engagement”, and showed that all three of three patients with large lesions went on to recurrence, despite arthroscopic Bankart repair (Fig. 2). Other clinical studies have corroborated this finding [16, 24].

The size of clinically relevant Hill Sachs lesions has not been clearly defined. Most anecdotal evidence suggests defects larger than 20 % of the humeral head require surgical management. One cadaveric study found that osteotomies occurring at 5/8 radius (approximately 38 % defect) initiated significant instability in positions of function [12]. Another study had the same result with a 30 % head defect, but no episodes of instability with 15 % defects [25].

The concept of the glenoid track is again relevant when considering Hill Sachs lesions and joint stability [22, 23], as was mentioned earlier. Hill Sachs lesions must be considered in the context of anterior glenoid bone loss, as probability for recurrent instability increases with larger bony defects on either side of the joint. In addition, as was discussed by Giacomo et al. [23], location of the lesion plays a large role in determining stability. Medially based lesions are anatomically closer to the anterior glenoid rim, and are therefore more likely to engage during range of motion, thereby causing symptoms of recurrent instability.

### Capsular laxity

One of the most commonly cited errors with stabilization surgeries includes failure to recognize and address capsular laxity during repair [14]. With multiple shoulder dislocations, anterior capsular tissue may be stretched and become redundant. A biomechanical study by Bigliani et al. [26] demonstrated that anterior capsular stretching can occur with or without labral detachment. As a result, persistently lax capsular tissue may be responsible for failure, even after a Bankart repair. Rowe et al. [11] showed that 83 % of patients with recurrent dislocations after surgical repair had significant capsular laxity, with these lesions highly correlated with re-dislocation. Significant capsular redundancy was again noted in the majority of failures by Meehan et al. [27] and Marquardt [20] .

Under-appreciation of humeral avulsions of the glenohumeral ligament (HAGL) is also responsible for persistent postoperative instability. Inability to correctly identify HAGL lesions on preoperative imaging or during intra-operative evaluation will inevitably lead to lack of proper treatment for this pathology. A high index of suspicion is necessary to identify and repair this lesion, which can appear in 9 % of anterior instability cases [28].

### Technical error

Meticulous attention to soft tissue tension and bony anatomy is essential for a favorable outcome. During Bankart repairs, suture anchors should be placed 1–2 mm onto the face of the articular glenoid rim, in order to adequately restore tension to the capsular tissue. Repairs focused medially on the glenoid neck fail to produce proper tension and functionally create ALPSA (anterior labroligamentous periosteal sleeve avulsion) lesions [9]. Particular attention should also be given to the level of the inferior most anchor. Anchors placed in a superior position fail to address laxity within the inferior capsule and inferior glenohumeral ligament [29, 30]. Number of anchors placed has also been implicated, with many failures resulting from fewer than three anchors [16, 24, 29]. Suture orientation, either in a simple or horizontal configuration, may also play a role in restoring native labral anatomy and improving stability [31]. Suboptimal results can also result from suture failure due to soft tissue cut-through or knot breakage/loosening [32].

Technical errors with coracoid bone transport procedures include block placement too medially (resulting in instability) or too laterally (arthrosis). Unicortical screws or insufficient graft compression can result in nonunion and breakage of screws [5, 33].

Many of the above factors can now easily be assessed via a preoperative score devised by Balg et al. [34], in order to determine potential risk for recurrence, particularly after arthroscopic repairs. This ten point score incorporates factors such as age, sports participation, hyperlaxity, and bony defects to determine efficacy of the procedure. Those scoring above six points had a high rate of recurrence, and were recommended for a bony transfer.

## Evaluation

When initially evaluating a patient who has failed surgical management for recurrent shoulder instability, a thorough clinical workup is necessary to determine the cause of failure. Proper diagnosis is the basis for identifying the appropriate surgical management. Careful analysis of the history, physical exam, and appropriate imaging will enable the clinician to avoid common pitfalls and optimize the chances for successful revision.

### History

Understanding the cause of failure after surgery can commonly be identified by the history. Inquiries into the nature of the “failure” are important, as some patients may complain of pain or stiffness rather than recurrent instability, all of which require different modes of treatment. In cases of instability, determining the circumstance that initiated episodes may be significant, as traumatic events resulting in dislocations suggest disruption of a previous Bankart repair. In contrast, smaller incidents associated with little to no trauma and mid-range symptoms of instability are indicative of missed, untreated capsular laxity from the index procedure or large bony defects in the glenoid or humeral head [35].

Patient factors such as age and co-morbidities should be noted. Younger patients have naturally more compliant tissue, and are therefore more likely to have a recurrent Bankart lesion. Older patients, particularly above 40 years of age, often have associated rotator cuff tears [36, 37]. Medical history, especially pertaining to patients with inherited collagen disorders like Ehlers-Danlos syndrome, present a unique challenge for treatment. Their generalized laxity often requires special attention, as standard treatment options are often insufficient.

Clinical reports from prior procedures should always be obtained, not only to determine the type of procedure performed, but also to elucidate other findings during the surgery. This may include evaluation of bony defects, capsular thinning, and other associated injuries such as rotator cuff or SLAP tears [33]. If an open Bankart procedure was performed, a failed subscapularis repair may be the etiology for symptoms.

### Physical exam

The physical exam should focus on range of motion, strength, stability, and laxity testing, with comparisons to the contra-lateral side. Range of motion should assess flexion, abduction, internal rotation, external rotation, and external rotation at 90 degrees of abduction in order to identify potential stiffness. In addition, excessive external rotation may suggest a subscapularis tear or redundant anterior capsule. Strength testing should particularly evaluate the rotator cuff, as concomitant tears are not infrequently associated [37]. Particular attention to the subscapularis muscle function is warranted after a failed open Bankart procedure, with assessment of a belly press and lift-off test [27].

Shoulder stability is best assessed with the apprehension test, followed by the relocation test. Load and shift tests can be attempted in the office; however, patients may be too guarded to allow a reliable exam.

Ligamentous laxity testing is performed to assess for both inherited (i.e., collagen disorders) as well as acquired (i.e., traumatic etiology) capsular redundancy. The sulcus sign, involving traction of the arm inferiorly, is present when dimpling of the skin occurs between the acromion and humeral head, indicating inferior laxity. The hyperabduction test, involving passive glenohumeral abduction greater than 105 degrees, is also indicative of inferior glenohumeral ligament dysfunction [38]. Finally, generalized laxity can be assessed by use of the Beighton score [39]. As previously mentioned, diagnosis of capsular laxity (either inherited or acquired) is imperative, as this may alter surgical treatment and prognosis.

### Imaging

Imaging is essential for the evaluation of patients with recurrent instability since it allows for the identification and quantification of glenoid bone loss. Standard radiographs are often the first mode of testing, due to accessibility. This should include a standard AP, true AP, and axillary views. The West Point view may also be considered for further assessment and has been found to be more sensitive for depicting bony lesions when compared to standard axillary views [40]. Accuracy of radiographs, in general, can be highly dependent on patient positioning, and often can only be suggestive of bone loss [35].

Computed tomography (CT) scans, on the other hand, provide a more reliable and detailed assessment of glenoid bone deficiency, and have become essential during preoperative planning. More recently, three-dimensional (3D) CT scans have been increasingly employed, and have been shown to be more accurate and effective than two-dimensional (2D) scans. A laboratory study by Bois et al. [41], utilizing saw bones, demonstrated the superiority of 3D models in predicting bone loss when compared to 2D studies, with equivalent reliability. These findings were corroborated by cadaveric studies that found 3D CT scans to be more accurate than radiographs, 2D CT scans, and MRI, when evaluating for bone loss [42, 43]. In a study looking at the utility of 3D scans in determining operative management, Chuang et al. [44] found a 96 % correlation between 3D CT scan measurements and arthroscopic evaluation. They concluded that CT scan utilization is an effective preoperative tool.

Evaluation of Hill Sachs lesions may be more difficult. In a radiographic study, Osaki et al. [45] detected only 90 of 118 lesions using CT imaging. Assessment of width and depth of such lesions using 2D CT has had good results, but further studies need to be done to improve these measurements [46].

MRI arthrograms have a limited role in evaluating instability. This modality enables the surgeon to confirm capsulolabral pathology and evaluate for other soft-tissue injury, such as a SLAP or rotator cuff tear. Though not as accurate as CT imaging for bony defects, MRI arthrograms are still a better predictor of lesions than standard radiographs.

### Intraoperative evaluation

Clinical examination of the shoulder, including load and shift testing, should be conducted once a patient is adequately anesthetized, confirming the degree of laxity found in the clinic. Often times, load and shift testing under sedation can demonstrate greater laxity than what was observed in the clinic setting.

Diagnostic arthroscopy should always be performed, as this modality enables direct evaluation of soft tissue lesions (Bankart lesion, HAGL lesion, capsular laxity, tissue quality [47]) as well as bony defects (anterior glenoid fracture/erosion, engaging Hill Sachs lesions [48]). Anterior bone loss can be determined intra-operatively by the bare spot method described by Burkhart and De Beer [9]. Hill Sachs depth and width as well as engagement in abduction and external rotation can also be confirmed.

## Management

When compared to index procedures, increased recurrence rates and poorer outcomes can be expected after revision surgery [20, 49]. Surgical management is also complicated by altered native anatomy from prior exposures, as well as previous placement of hardware [50].

Fundamental to surgical success is determining the cause of the previous procedure’s failure. Misdiagnosis with untreated pathology must be identified and corrected with the following revision; otherwise, repeat recurrence can be expected.

### Non-operative management

Non-operative management should consist of immobilization, followed by gradual physical therapy and strengthening for at least 6 weeks [7]. Patients need to be educated on avoiding at-risk arm positions [51]. Following rehab, some patients may be satisfied with their postreduction result and prefer continuing with non-operative management, despite having one or more episodes of recurrent instability [5].

### Revision bankart repair

In cases with anterior instability associated with a detached capsulolabral complex and minimal glenoid deficiency (less than 25 %), revision Bankart repair is indicated. Open Bankart repair has been the gold standard, with recent studies showing recurrence rates ranging from 0 to 13 %, after revision surgery [7, 50, 52–54] (Table 2). A recent trend has shifted interest in performing arthroscopic repairs. Revision operations with modern implant designs and techniques have resulted in recurrence rates ranging from 6 % to 27 % in small case series [19, 29, 30, 49, 55–58] (Table 3). Since similar success has been shown with either modality, revision surgery for a classic Bankart lesion can be performed with either technique, with expectation of good to excellent results.

**Table 2:** Recurrence rates after revision open Bankart repairs

Study	*n*	Mean follow up (months)	Recurrence rate (%)
Sisto et al. [7]	30	46	0
Friedman et al. [50]	73	44.2	5.5
Araghi et al. [52]	23	–	9
Cho et al. [53]	26	42	11.5
Neviaser et al. [54]	30	122	0

**Table 3:** Recurrence rates after revision arthroscopic Bankart repairs

Study	*n*	Mean follow up (months)	Recurrence rate (%)
Arce et al. [19]	16	30.9	18.8
Bartl et al. [29]	56	37	11
Shin et al. [30]	63	46.9	19.0
Krueger et al. [49]	20	25	10
Neri et al. [55]	11	34.4	27
Patel et al. [56]	40	36	10
Barnes et al. [57]	17	38	5.9
Abouali et al. [58]	349	35.4	12.7

Special attention should be given to patients presenting with capsular redundancy, with or without a Bankart lesion. Failure to recognize and treat this pathology may result in persistent laxity, with subsequent failure. A laterally based open capsular shift, as described by Neer and Foster [59], has been shown to be effective. In five patients with capsular laxity and a positive sulcus sign, a T-type capsular shift resulted in no recurrences [14]. Much like with Bankart repairs, this type of procedure has given way to the more popular, arthroscopic version [60]. Arthroscopic capsular plication involves moving the capsule sequentially from inferior to superior onto the glenoid face, thereby tightening the patulous inferior capsule [61]. Further tension on the redundant tissue can be restored by performing rotator interval closures [7, 33].

### Capsular repair and reconstruction

Significant capsular laxity with inadequate, poor quality, or deficient tissue is uncommon, but can be attributed to multiple, failed procedures or iatrogenic causes such as thermal capsulorraphy. Many of these patients are naturally predisposed to this condition due to an underlying connective tissue disorder, such as Ehlers-Danlos syndrome. Such deficiency is a challenging problem, particularly in the young patient. Treatment options are limited, and include revision reconstruction and glenohumeral arthrodesis [47]. Reconstruction includes autograft options with hamstring tendon [62, 63] or iliotibial band [64] as well as allograft options [65]. More recently, Dewing et al. [47] published results on salvage reconstruction in 20 shoulders, using either a tibialis anterior allograft or semitendinosus autograft. At mean follow up of 3.2 years, 6 of 20 shoulders required further surgery for persistent instability, with an additional 3 shoulders progressing to surgery for pain. Though these findings illustrate the difficulty with managing this complex problem, over half of the patients were able to maintain stability.

### Glenoid bone augmentation

**Fig. 3:**
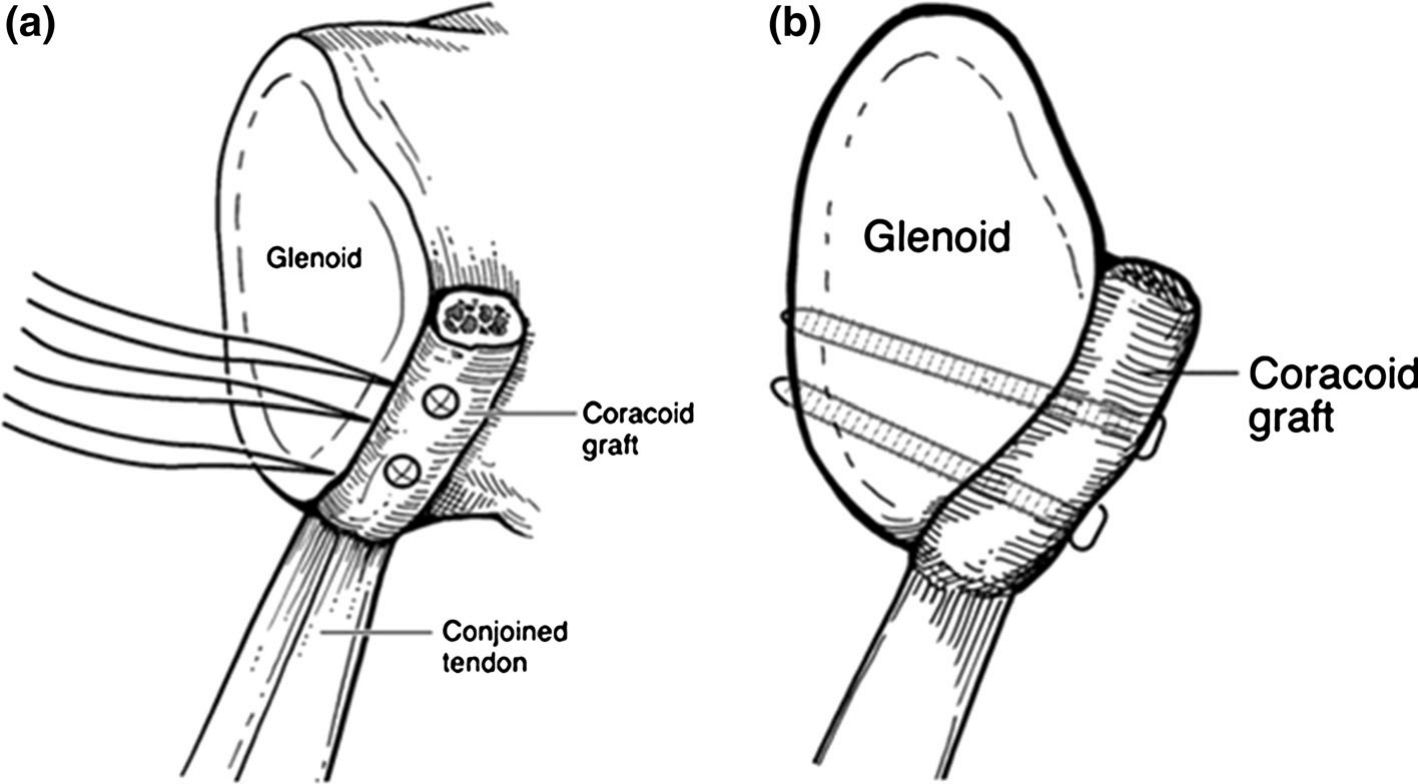
Schematic of bony block transfer procedure looking from anteriorly (**a**) and laterally (**b**), with transfer of the coracoid tip and soft tissue attachments to the anterior glenoid rim [9]

The mainstay treatment for glenoid bone loss greater than 25 % is a Bristow or Latarjet procedure (Fig. 3), involving transfer of the coracoid bone block to the anterior aspect of the glenoid. This technique provides stability via three mechanisms: extension of the glenoid’s bony articular arc, tethering effect from the transferred conjoined tendon, and repair of the anterior capsule to the coraco-acromial ligament. Burkhart and De Beer [66] showed a 60 % recurrence rate in shoulders with bone loss that were stabilized with only a soft tissue repair, with reduction to 5 % recurrence in the same category of patients after an open modified Latarjet. Similar results have been noted with arthroscopic Bristow-Latarjet repairs [67].

Another method for restoring the bony articular arc is through augmentation with autologous tricortical iliac crest bone graft. In two clinical studies, open techniques showed no recurrences with graft union in all patients [35, 68]. Arthroscopic techniques for graft fixation have also been described [69].

### Hill sachs management

Hill Sachs lesions with a defect larger than 20–25 % of the humeral head are generally recommended for surgical intervention, whereas those less than 20 % can reliably be stabilized with an isolated Bankart repair. Treatments for Hill Sachs lesions can be subdivided into anatomic and non-anatomic procedures.

Anatomic procedures attempt to restore proximal humerus bone loss by filling the defect. Humeral head reconstruction with osteochondral allograft transplantation is effective for large lesions and can use femoral head or humeral head allograft, with or without screws. This procedure may be associated with high complication, re-operation, and resorption rates [70]. Defects can also be filled via a transhumeral approach that involves utilizing an anterior cruciate ligament tibial guide and bone tamp to localize and elevate the subchondral bone with allograft bone chips [71].

**Fig. 4:**
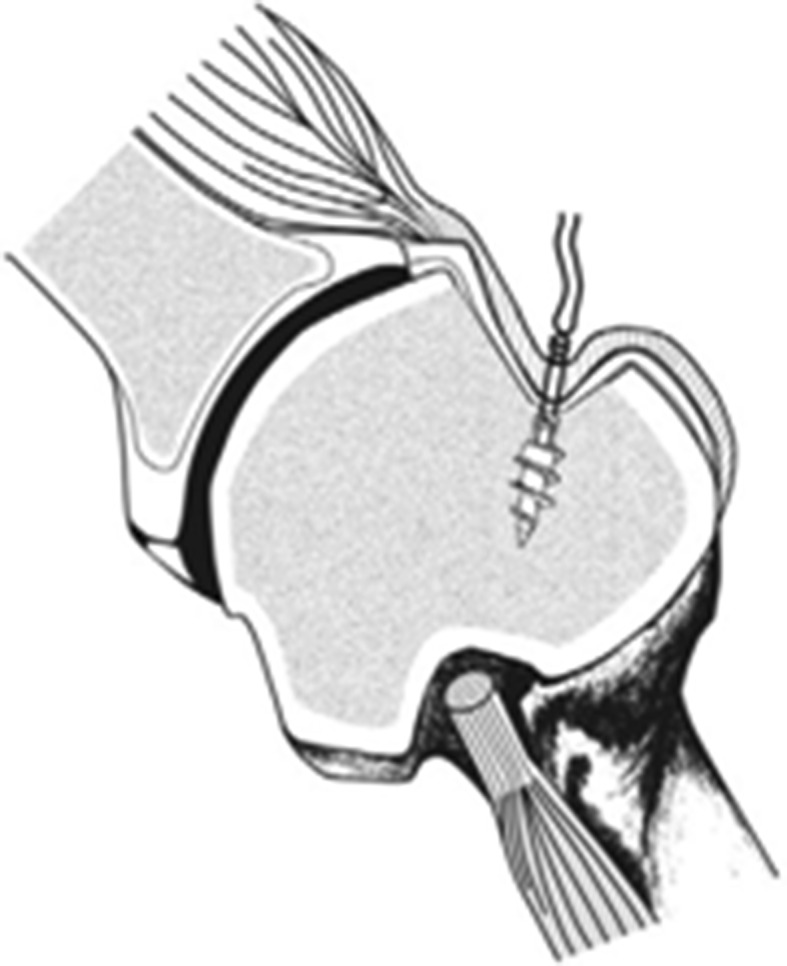
Remplissage technique, with posterior capsulotenodesis into the Hill Sachs defect [80]

Non-anatomic procedures aim to alter anatomy in order to prevent engagement of the Hill-Sachs lesion. Popular techniques include the remplissage and Latarjet. The remplissage procedure, meaning “to fill” in French, involves capsulotenodesis of the posterior capsule and infraspinatus tendon into the defect (Fig. 4). This technique renders the defect extra-articular and tightens the posterior restraints, functionally preventing engagement and acting as a check-rein against anterior translation. It should not be performed in isolation, but should be used to augment a Bankart repair. Arthroscopic techniques have been described with excellent and durable results, reliable healing, and minimal loss of motion, even in the revision setting [48, 72]. In a recent study by Cho et al. [73], significant increases in recurrence rates were noted in patients with engaging Hill Sachs lesions who received only a Bankart repair (26 %), compared to those with a combined Bankart repair with remplissage (5.4 %). The Latarjet procedure is also effective in preventing recurrences by extending the glenoid arc via transfer of the coracoid process. Biomechanical and clinical studies show that the Latarjet confers equivalent stability when compared to the remplissage [74], but it may be associated with higher complication rates [75].

### Failed coracoid bone transfer (Latarjet/Bristow procedure)

Severity of bone loss must be determined before planning of revision surgery.

If a patient had undergone the initial coracoid transfer in the setting of *minimal or no* bone loss, and more recent imaging confirms preserved glenoid bone stock, revision Bankart repair (either arthroscopic or open) may be indicated. This involves labral reattachment (or capsular reattachment when the labrum has been resected) using suture anchors, with or without capsular plication. Boileau et al. [33] found high satisfaction rates with revision arthroscopic Bankart repairs following failed open procedures (the majority of which were Latarjets). Castagna et al. [76] had a 16.7 % rate of recurrence after revision arthroscopic treatment. Revision open Bankart repairs have also been described, and are an option in more complex cases [27, 54].

On the other hand, if the patient underwent the initial coracoid transfer due to *significant* glenoid bone loss, an isolated Bankart repair can be expected to be insufficient. In these more complicated cases, reconstruction of the bone deficit is necessary, using either autograft or allograft bone, with subsequent repair of the capsule to the graft. For reconstitution of the glenoid arc, autograft options include iliac crest [35] or distal clavicle [77], while allograft sources include distal tibia [78] or iliac crest [79].

## Conclusion

Treatment of shoulders that have failed a stabilization procedure can be a challenging task. Identifying risks factors such as age and chronicity of instability is important, as this information can be predictive of future stabilization outcomes. More importantly, evaluation of the cause for previous post-surgical failure is crucial. Common mistakes include missed diagnoses or under-correction of bony defects in the glenoid or humeral head, technical errors with suture anchor placement, and unaddressed capsular laxity. Treatment plans should then aim to address these deficiencies. Simple, recurrent Bankart lesions can be treated with either an arthroscopic or open Bankart repair, whereas capsular deficiency requires more complex treatment such as reconstruction. Glenoid bone defects are most appropriately treated with either bone grafting or coracoid transfer, while large Hill Sachs lesions can be addressed with a remplissage, Latarjet, or bone grafting.
